# Supplementary material for: Enhancing Anti-Tumorigenic Efficacy of Eugenol in Human Colon Cancer Cells Using Enzyme-Responsive Nanoparticles
Source: Cancers (Basel). 2023 Feb 10;15(4):1145. doi: 10.3390/cancers15041145 (PMC9953800; doi:10.3390/cancers15041145)
Supplement: Supplementary file 1 [file cancers-15-01145-s001.zip › cancers-2193321-supplementary.pdf]

Supplementary Materials

Name	Manufacturer and Catalog number	Dilution	Catalog number
IRDye 800CW Goat anti-Rabbit IgG (H+L),	LI-COR Corporate	1:3000	827-08365
925-68020 IRDye® 680LT Goat anti-Mouse IgG (H + L)	LI-COR Corporate	1:3000	925-68020
Human BD Fc Block	BD Pharmingen	1 µl/10^6 cells	564220
Ms IgG2b Kpa PE	BD Pharmingen	1 µl/10^6 cells	555743
PE Mouse Anti-Human CD44	BD Pharmingen	1 µl/10^6 cells	561858
APC Mouse Anti-Human CD133	BD Pharmingen	1 µl/10^6 cells	566597
APC Mouse IgG1 k Isotype Control	BD Pharmingen	1 µl/10^6 cells	554681
Alexa Flour 647 Rat IgG2b, k Isotype Control	BD Pharmingen	1 µl/10^6 cells	557691

Table S1. Antibodies and their dilutions used in this study.

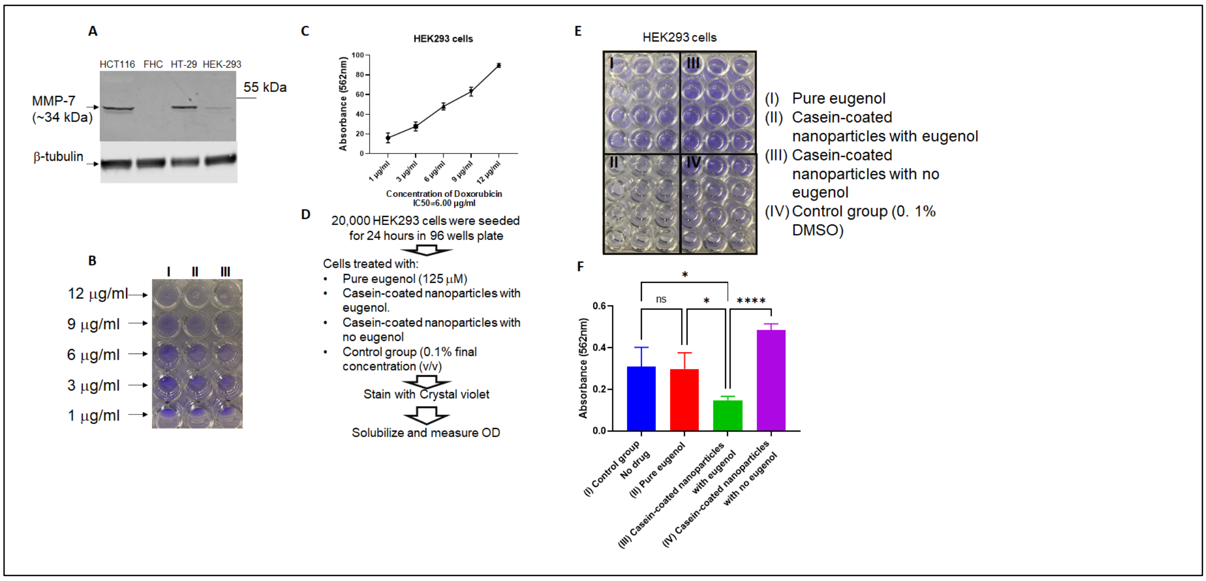

Figure S1. Casein-coated nanoparticles with EUG as opposed to pure EUG induce cytotoxicity in the human embryonic kidney (HEK)-293 cells. (A) Anti-MMP-7 shows high expression of MMP-7 in colon cancer cells, while FHC cells showed no trace of intracellular MMP-7. HT-29, a colon cancer cell line with dominant metastatic characters similarly showed an enriched MMP-7 cytoplasmic pool (a positive control for HCT-116 cell line). HEK293 cells showed a trace of MMP-7. To validate the accuracy of the crystal violet assay (#ab232855), HEK-293 cells were treated with different concentrations of doxorubicin (DOX) for 24 hours, according to the kit protocol in triplicate (B). DOX as a cytotoxic molecule, inhibits cell growth. Panel C shows the dose-response absorbance (%) of DOX determined by the absorbance (562 nm wavelength) of remaining stained cells. Panel C confirmed that the absorbance

values at 562 nm are directly proportional to the remaining cells after DOX treatment in the crystal violet staining assay. Panel D illustrates the steps of the crystal violet assay conducted for HEK-293 cells (Panel D) in comparison to FHC and HCT-116 cells (Main Figure 10). Panels E and F show that pure EUG (125µM) failed to induce toxicity in HEK-293 cells after 48 hours (red column versus blue control column). However, EUG (125µM) delivered by NPs significantly decreased the remaining attached cells (green column versus blue and red columns). HEK-293 cells treated with casein-coated NPs with no EUG showed a significant elevation of stained cells (purple column).

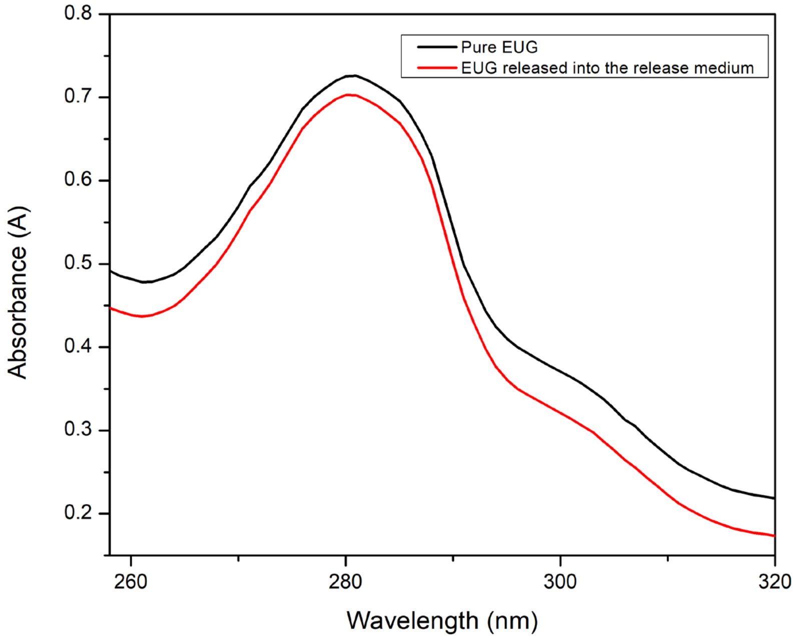

Figure S2. Chemical Stability of Eugenol. UV spectra of eugenol solution at pH 7.4 before and after exposure to the drug-release medium for 5 days. showing the chemical stability of Eugenol in its pure solution and released medium at pH 7.4. There are no noticeable differences in the obtained absorbance spectra., suggesting the chemical stability of the eugenol during nanoparticle under loading and drug release conditions.

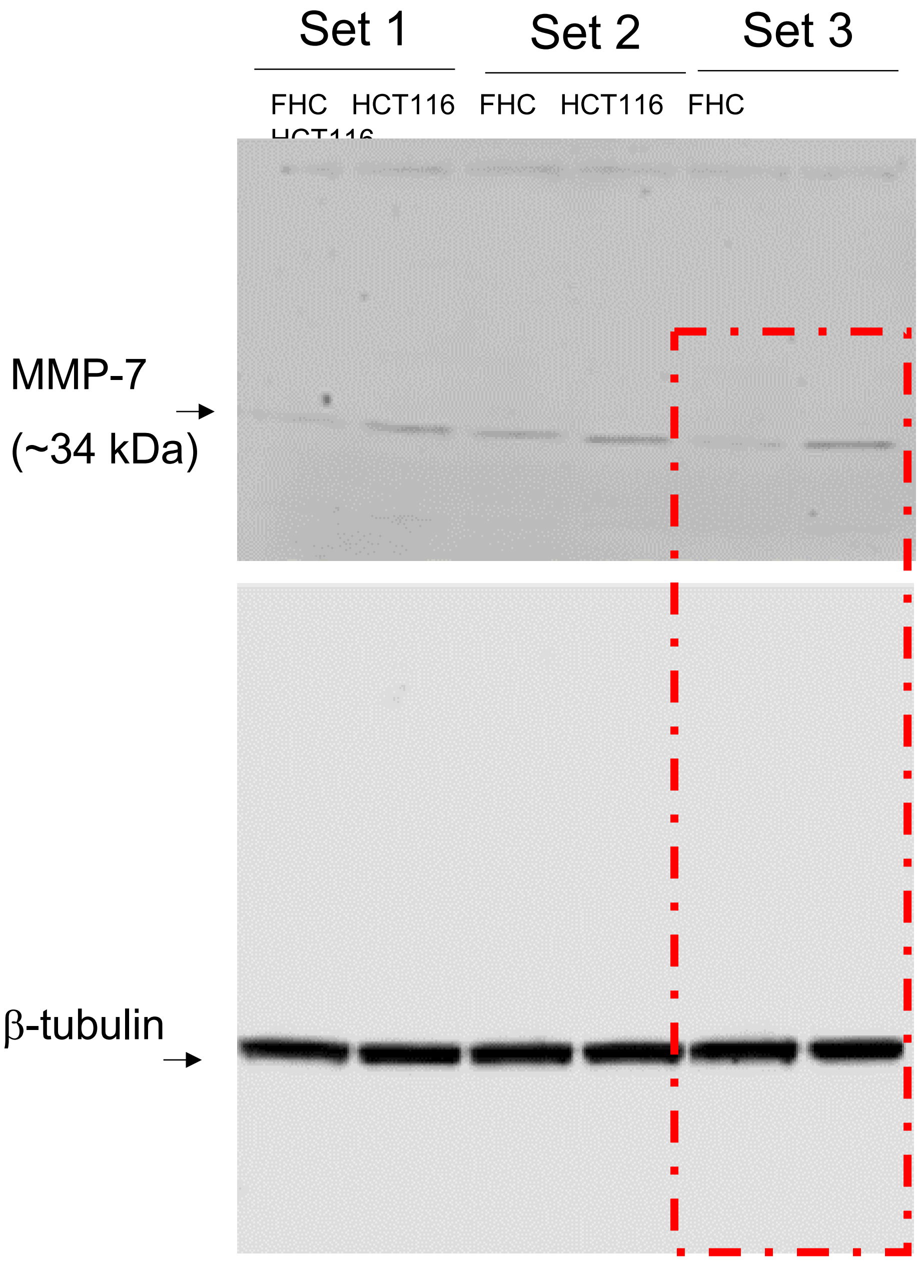
Set 1
Set 2
Set 3
FHC HCT116 FHC HCT116 FHC
HCT116
MMP-7
(~34 kDa)
β-tubulin

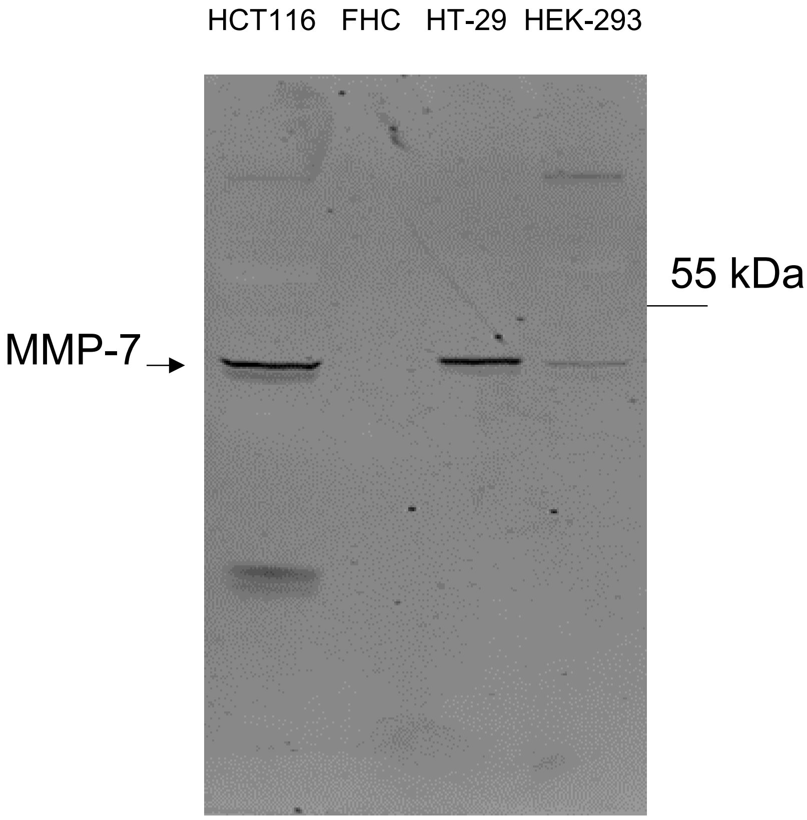

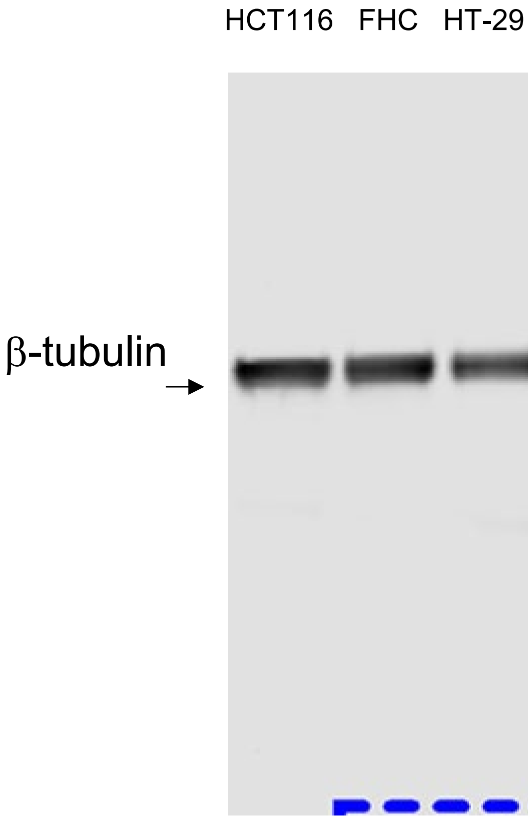

Figure S3. The whole western blots
